# Radiation Shielding Evaluation of Carbohydrate Hydrogel Radiotherapy Pads Containing High-Z Fillers: A Geant4 Study

**DOI:** 10.3390/polym17162234

**Published:** 2025-08-17

**Authors:** Hanan Akhdar, Samar Alghamdi

**Affiliations:** 1 Department of Physics, Faculty of Science, Imam Mohammad Ibn Saud Islamic University (IMSIU), P.O. Box 90950, Riyadh 11623, Saudi Arabia; 2 Basic Sciences Research Center (BSRC), Deanship of Scientific Research, Imam Mohammad Ibn Saud Islamic University (IMSIU), Riyadh 11623, Saudi Arabia; samar.awwad.alghamdi@gmail.com

**Keywords:** carbohydrate hydrogels, radiotherapy pads, Geant4

## Abstract

This work analyzes the radiation shielding effectiveness of biocompatible hydrogel pads containing carbohydrate-based polymer matrices (Alginate, Chitosan, and Cellulose) integrated with the high atomic number (Z) fillers Bismuth Oxide (Bi_2_O_3_) and Zinc Oxide (ZnO). The Monte Carlo-based toolkit, Geant4, was used to simulate the deposition of the dose throughout a multilayer phantom that mimics the skin (Epidermis, Dermis, Subcutaneous, and Muscle) with a pad on top irradiated with photon and electron beams from 50 keV to 1000 keV. The results indicated that Bi_2_O_3_ succeeded in causing greater absorption of photons at doses, particularly in deep-layer tissues, from the increase in the filler content as well as the pad thickness. The Cellulose–Bi_2_O_3_ composites (10 mm thick) not only showed the best deep-shielding property among all investigated combinations but also the Alginate-based pads generally performed better with regard to the surface dose attenuation. The results demonstrate the promising potential of high-Z-doped hydrogels in serving as flexible, light, and biocompatible shielding materials for superficial radiotherapy.

## 1. Introduction

Minimizing the undesired radiation exposure to healthy tissue in superficial radiotherapy remains a substantial challenge, for which traditional shielding materials are frequently inadequate in view of their rigidity, weight, and degree of biocompatibility. Novel biomaterials, including Alginate, Chitosan, and Cellulose, among others, demonstrate potential as they are hydrophilic and medically safe [1,2,3,4,5]. Unfortunately, such polymers are only partially effective in attenuating ionizing radiation because of their low atomic numbers (Z). To overcome this drawback, the loading of fillers of high-Z elements such as Bismuth Oxide (Bi_2_O_3_) and Zinc Oxide (ZnO) may be an effective means to improve their shielding capabilities [6,7,8]. Such composite pads could potentially be used as patient-specific, user-friendly shielding covers during radiation therapy, particularly in the case of patients who need to shield a region of interest that is sensitive or exposed. For this purpose, we use Geant4, an advanced Monte Carlo simulation toolkit, to simulate the transport and energy deposition of radiation through multi-layered tissue-equivalent geometries [9,10,11]. The dose reduction performance of polymer pads containing bismuth oxide (Bi_2_O_3_) and zinc oxide (ZnO) is systematically explored as a function of thickness and filler percentage. The purpose is to quantitatively evaluate the shielding capability of the hydrogel composites and to confirm their performance as radiotherapy pads for clinical use.

## 2. Materials and Methods

### 2.1. Overview

In this work, a Geant4 (version 11.03) Monte Carlo simulation was used to estimate the radiation shielding performance of Alginate, Chitosan, and Cellulose-based hydrogel radiotherapy pads with and without high-Z dopants (Bi_2_O_3_ and ZnO) [10]. The energy deposition was studied in a multilayer tissue-equivalent phantom when irradiated by monoenergetic photon and electron beams with energies from 50 keV to 1000 keV. The effect on energy deposition in individual tissue layers was investigated by varying key parameters, including pad material, thickness (2 mm, 10 mm), and filler concentration (0%, 5%, 10% by weight).

In order to provide indirect validation of the present Geant4 model, our results were compared with available experimental and Monte Carlo–validated data from the literature. The increased low-energy photon attenuation observed for Bi_2_O_3_-doped pads is in agreement with published experimental work, which reported similar enhancement factors for Bi_2_O_3_-loaded polymer matrices [7,8]. The moderate but consistent photon and electron shielding performance of ZnO-doped pads in our simulations matches the experimental trends observed for ZnO/polymer composites in a previously published article [9]. Our depth–dose distributions in multi-layer tissue phantoms also align with those measured in slab phantom experiments [12]. Such consistency with benchmarks based on the literature supports the reliability of the present simulation approach.

### 2.2. Simulation Geometry and Phantom Construction

The world volume of the simulation setting was a 20 × 20 × 20 cm^3^ water-filled container using the Geant4 pre-defined material (G4_WATER). All simulated structures were oriented along the z-axis, in order for radiation to enter the skin vertically.

**Figure 1:**
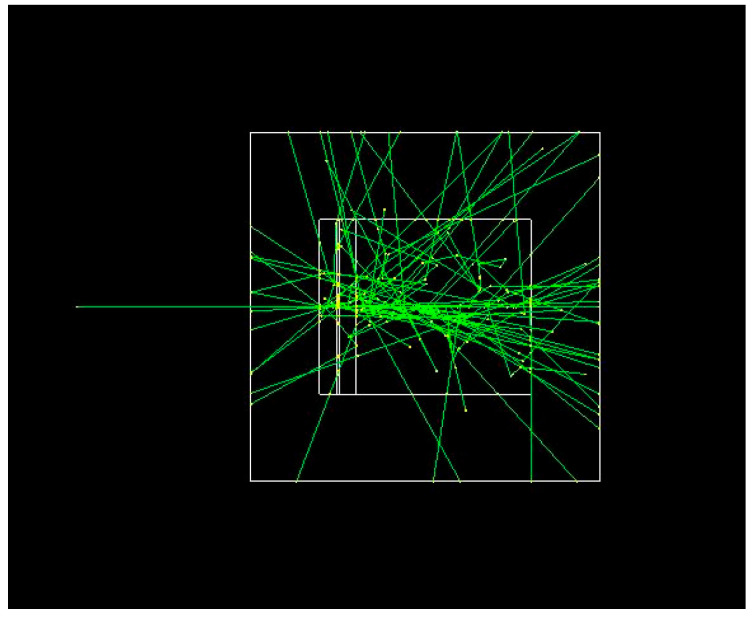
Visualization of the developed Geant4 simulation showing interactions between a monoenergetic beam (photon or electron) and a layered water-based tissue phantom. The phantom consists of stacked volumes representing skin, subcutaneous tissue, and muscle, with a hydrogel-based polymer pad positioned on top. The simulation was run with 1,000,000 events per beam type/energy.

The phantom, as shown in Figure 1, was built with a slab model of four layers modeled as a G4Box solid and positioned in depth: hydrogel pad, with a thickness of 2 mm, 10 mm; skin, with a 0.1-mm-thick epidermis using the Geant4 pre-defined material (G4_SKIN_ICRP); the dermis with a thickness of 1.0 mm using the Geant4 pre-defined material (G4_TISSUE_SOFT_ICRP); subcutaneous fat, 10 mm thick (G4_ADIPOSE_TISSUE_ICRP); and muscle with thickness of 100 mm using the Geant4 pre-defined material (G4_MUSCLE_SKELETAL_ICRP). Those layers are simplified but realistic and clinically represent a slab model comprising four tissue-equivalent layers to evaluate radiation interaction behavior [13,14,15].

Every hydrogel material was defined as a hydrated composite of 80% water and 20% dry polymer. Compositions and densities were chosen to be close to literature values of realistic hydrogel polymers.

A total of 1,000,000 primary events per configuration was used, following established practice in similar Geant4 dosimetry studies where this number ensures relative uncertainties for layer-averaged energy deposition that are typically ≤1–2% [16,17]. Given the relatively large scoring volumes, statistical noise was negligible compared to the magnitude of the reported effects.

### 2.3. Hydrogel Pad Material Definitions

#### 2.3.1. Base Hydrogel Composition

Each hydrogel material was modeled as a hydrated polymer composite, comprising 80% water and 20% dry polymer by weight. Elemental definitions and densities were assigned based on literature values to ensure physicochemical realism and biological relevance for topical biomedical applications. Table 1 summarizes the key material properties [18,19,20,21,22,23,24,25,26,27,28,29,30,31].

Each hydrogel pad was simulated in both pure and filler-enhanced forms.

#### 2.3.2. High-Z Dopants

To increase interaction cross-sections for photon and electron radiation, selected hydrogel formulations were doped with high atomic number (high-Z) fillers. Table 2 presents the properties and intended function of each dopant [6,7,8].

Hydrogel materials were doped with 5% and 10% of these fillers by weight.

### 2.4. Beam Source and Irradiation Conditions

Five energy levels were used to simulate photon and electron monoenergetic beams: 50, 100, 300, 500, and 1000 keV. The beams were simulated perpendicular to the superior surface of the hydrogel pad. All phantom layers were defined as scoring volumes to score the energy deposited within. Every simulation was performed with a million primary events for a reasonable statistical precision of energy deposition values.

## 3. Results

The simulations examined the photon and electron shielding capability of the investigated hydrogel pads.

### 3.1. Energy Deposition in the Epidermis

#### 3.1.1. Gamma Irradiation

**Figure 2:**
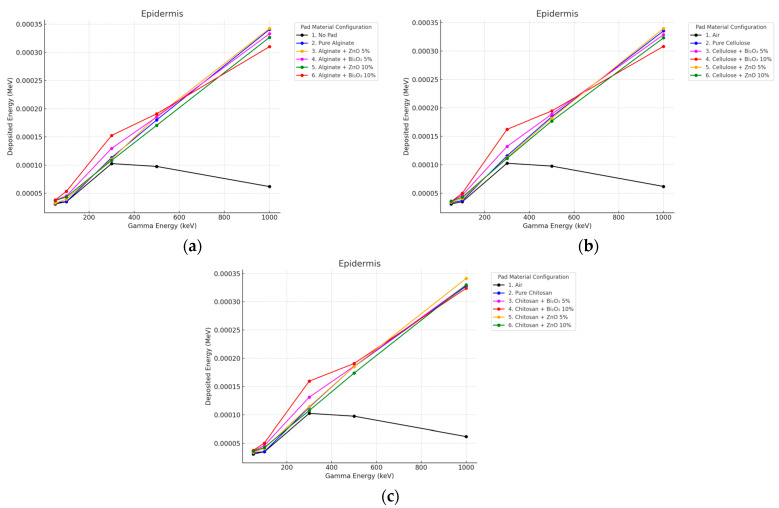
Photon energy deposition profiles in an (epidermis) under different polymer pad configurations at 2 mm thickness. (**a**) (Alginate-based). (**b**) (Cellulose-based). (**c**) (Chitosan-based).

**Figure 3:**
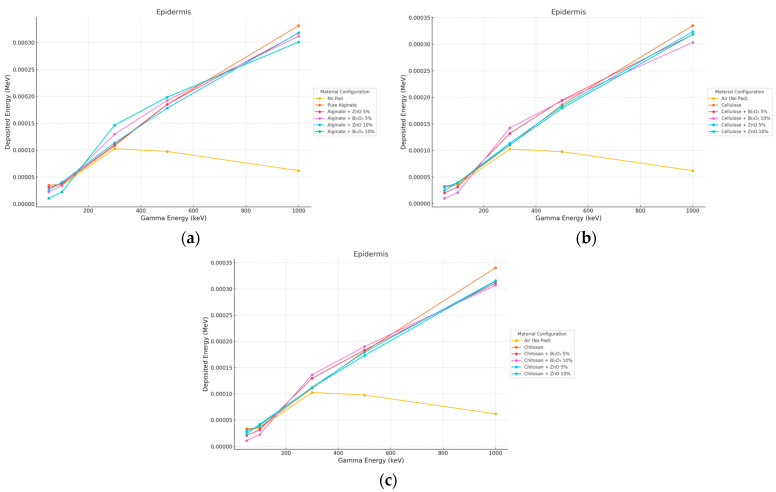
Photon energy deposition profiles in an (epidermis) under different polymer pad configurations at 10 mm thickness. (**a**) (Alginate-based). (**b**) (Cellulose-based). (**c**) (Chitosan-based).

The gamma beam energy deposition in the epidermis was found to greatly depend on the pad material and the content of fillers in the polymer as can be seen in Figure 2 and Figure 3. No Pad showed the least surface dose in all energies because of the probability of photons passing through and penetrating with less deposition energy. Bi_2_O_3_-doped, pads, especially at 10% concentration, showed the largest energy deposition in the epidermis in low photon energies (50–300 keV). This is due to the greatly Z^3^ dependence of the photoelectric effect. At 100 keV, the epidermal dose increased by 67% for Alginate + Bi_2_O_3_ 10% versus No Pad and 52% for Cellulose + Bi_2_O_3_ 10%. and 41% for Chitosan + Bi_2_O_3_ 10%.

#### 3.1.2. Electron Irradiation

**Figure 4:**
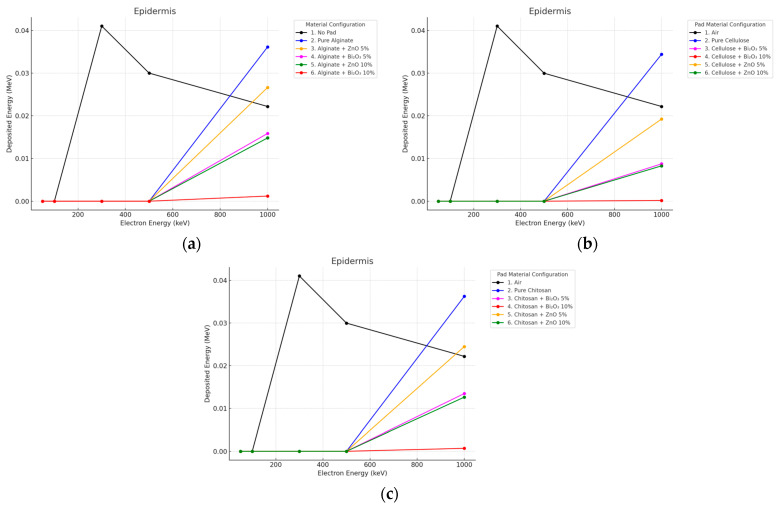
Electron energy deposition profiles in an (epidermis) under different polymer pad configurations at 2 mm thickness. (**a**) (Alginate-based). (**b**) (Cellulose-based). (**c**) (Chitosan-based).

**Figure 5:**
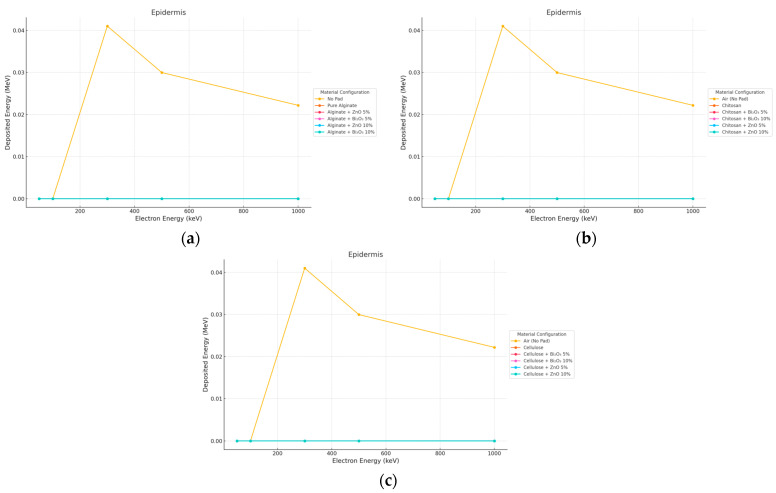
Electron energy deposition profiles in an (epidermis) under different polymer pad configurations at 10 mm thickness. (**a**) (Alginate-based). (**b**) (Cellulose-based). (**c**) (Chitosan-based).

The reverse behavior could be observed for electron beams as seen in Figure 4 and Figure 5. The highest epidermal dose was obtained with the No Pad set up for the maximum energy (300 keV) because of no attenuation of the electron beam and the characteristic Bragg peak near the surface. All hydrogel pads provided a significant reduction in surface dose. In the 10 mm thick pad case, all pads, doped and undoped, decreased the epidermal dose down to near zero levels (<0.00001 MeV). The surface dose was slightly increased in Bi_2_O_3_ 10% due to back-scattered electrons at a thickness of 2 mm, but still, the total energy deposition by the Bi_2_O_3_ pad at this thickness, compared to No Pad, exceeded 85%.

### 3.2. Energy Deposition in the Dermis

#### 3.2.1. Gamma Irradiation

**Figure 6:**
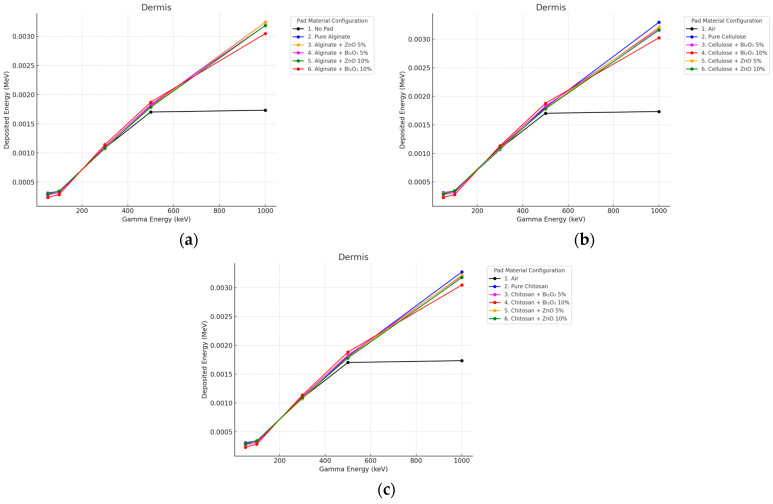
Photon energy deposition profiles in a (dermis) under different polymer pad configurations at 2 mm thickness. (**a**) (Alginate-based). (**b**) (Cellulose-based). (**c**) (Chitosan-based).

**Figure 7:**
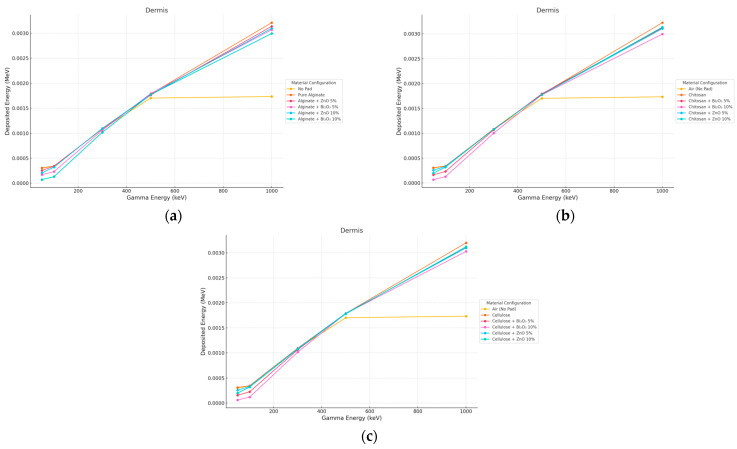
Photon energy deposition profiles in a (dermis) under different polymer pad configurations at 10 mm thickness. (**a**) (Alginate-based). (**b**) (Cellulose-based). (**c**) (Chitosan-based).

In the dermis, all pad geometries yielded a higher dose compared to air, especially for Bi_2_O_3_-contaminated samples as seen in Figure 6 and Figure 7. This is due to low energy electrons and photons generated within the pad that penetrate into mid-layers.

#### 3.2.2. Electron Irradiation

**Figure 8:**
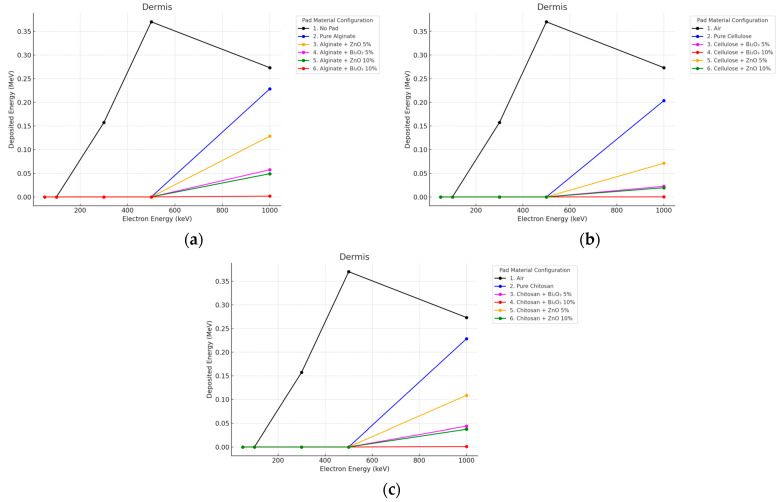
Electron energy deposition profiles in a (dermis) under different polymer pad configurations at 2 mm thickness. (**a**) (Alginate-based). (**b**) (Cellulose-based). (**c**) (Chitosan-based).

**Figure 9:**
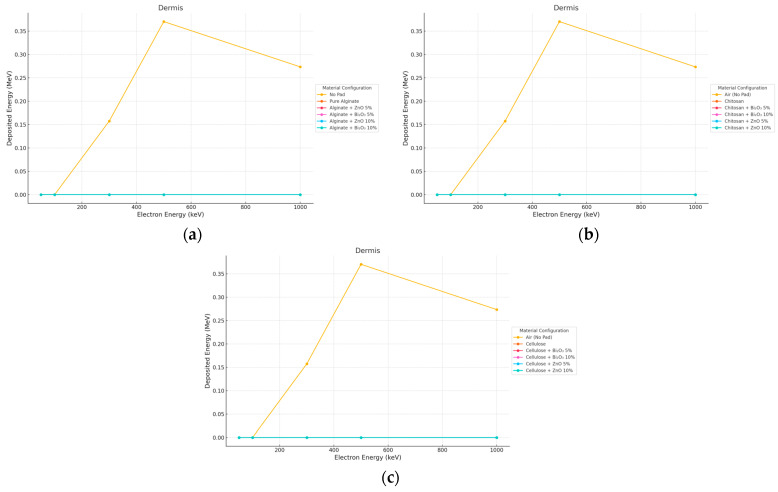
Electron energy deposition profiles in a (dermis) under different polymer pad configurations at 10 mm thickness. (**a**) (Alginate-based). (**b**) (Cellulose-based). (**c**) (Chitosan-based).

Electron beams were well attenuated in the dermis by the use of 10 mm thick pads as seen in Figure 8 and Figure 9.

### 3.3. Subcutaneous Layer Deposition of Energy

#### 3.3.1. Gamma Irradiation

**Figure 10:**
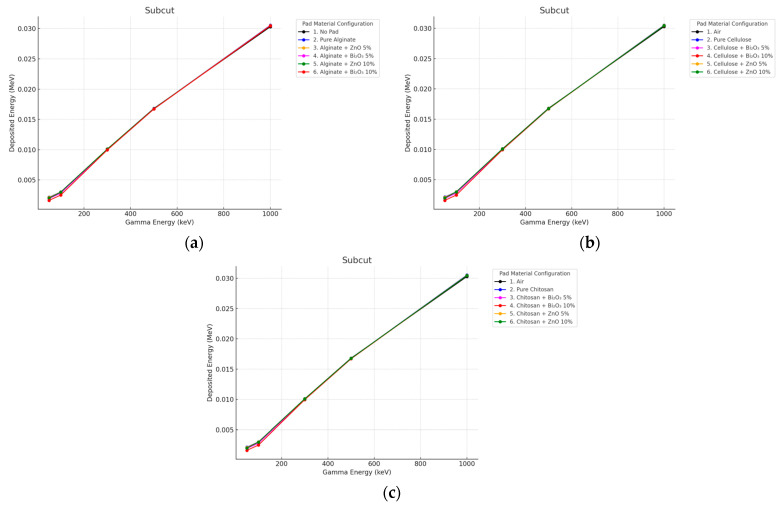
Photon energy deposition profiles in a (subcut) under different polymer pad configurations at 2 mm thickness. (**a**) (Alginate-based). (**b**) (Cellulose-based). (**c**) (Chitosan-based).

**Figure 11:**
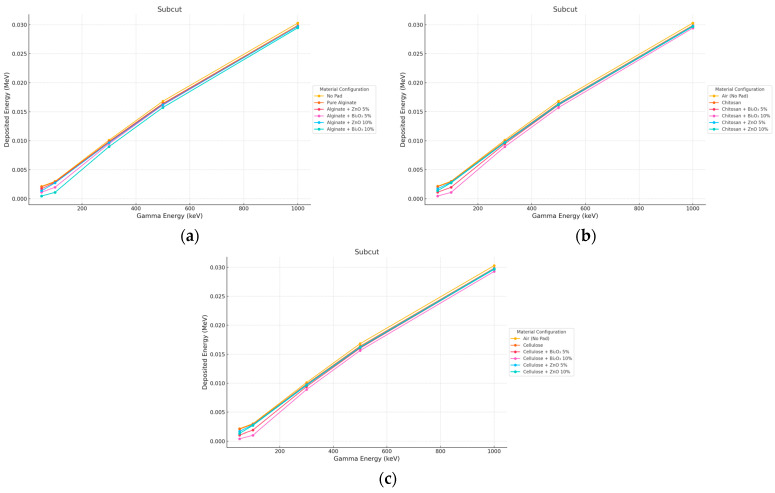
Photon energy deposition profiles in a (subcut) under different polymer pad configurations at 10 mm thickness. (**a**) (Alginate-based). (**b**) (Cellulose-based). (**c**) (Chitosan-based).

Minimal subcut influence from the pad was observed at either thickness. All curves, i.e., air, pure, and doped pads, nearly overlapped, particularly above 300 keV as seen in Figure 10 and Figure 11. This demonstrates that the attenuation of high-energy photons in deep tissues by thin (even 10 mm) pads is not an effective approach.

#### 3.3.2. Electron Irradiation

**Figure 12:**
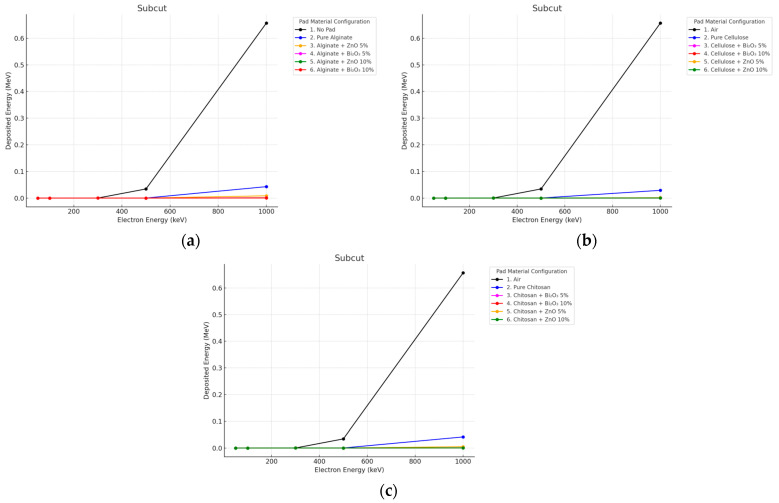
Electron energy deposition profiles in a (subcut) under different polymer pad configurations at 2 mm thickness. (**a**) (Alginate-based). (**b**) (Cellulose-based). (**c**) (Chitosan-based).

**Figure 13:**
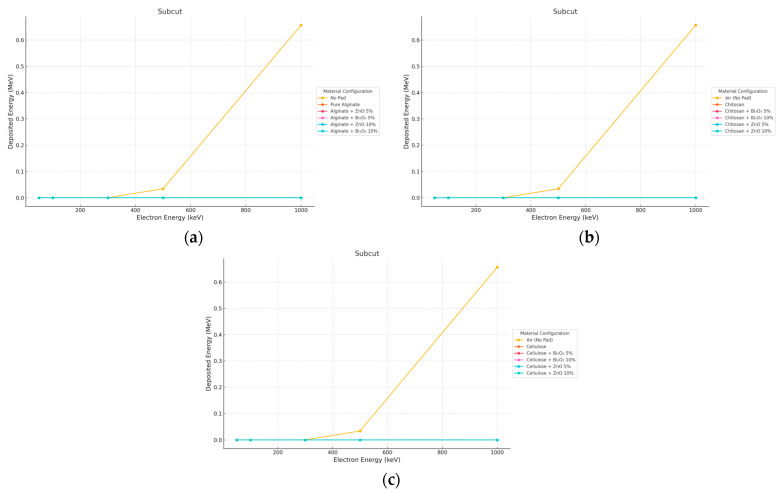
Electron energy deposition profiles in a (subcut) under different polymer pad configurations at 10 mm thickness. (**a**) (Alginate-based). (**b**) (Cellulose-based). (**c**) (Chitosan-based).

With 10 mm pads, the electron beams were heavily attenuated before they reached the subcut layer; doses decreased from ~0.65 MeV (No Pad) to <0.005 MeV in all shielded setups as shown in Figure 12 and Figure 13.

### 3.4. Energy Deposition in Muscle Layer

#### 3.4.1. Gamma Irradiation

**Figure 14:**
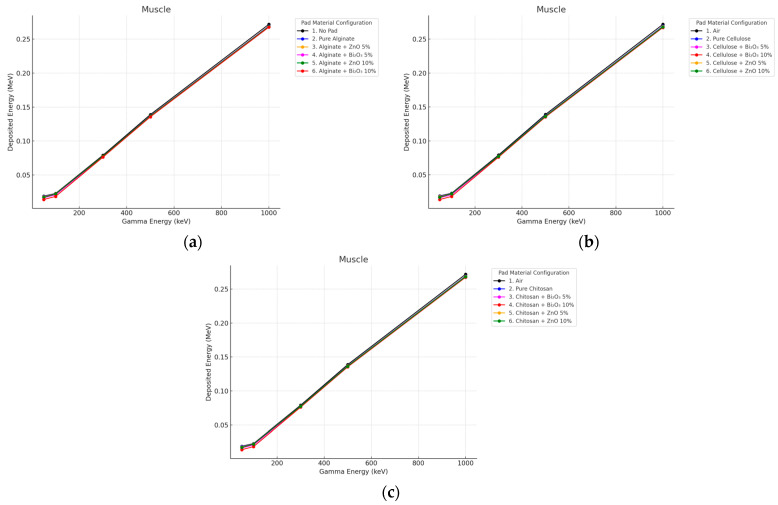
Photon energy deposition profiles in a (muscle) under different polymer pad configurations at 2 mm thickness. (**a**) (Alginate-based). (**b**) (Cellulose-based). (**c**) (Chitosan-based).

**Figure 15:**
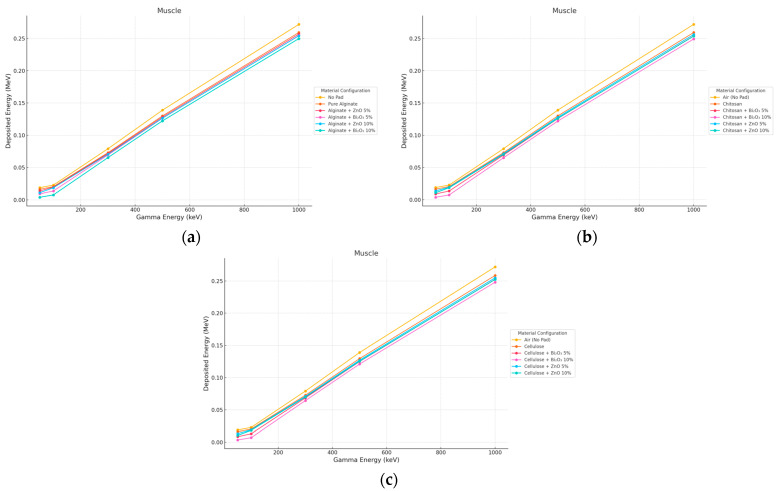
Photon energy deposition profiles in a (muscle) under different polymer pad configurations at 10 mm thickness. (**a**) (Alginate-based). (**b**) (Cellulose-based). (**c**) (Chitosan-based).

Gamma reached the muscular layer relatively uninhibited by the pad covering materials. At 1000 keV, the energy deposition in all configurations was within ±10% of No Pad. Cellulose + Bi_2_O_3_ 10% exhibited the best-defined attenuation, mainly in low energies (≤300 keV), related to increased surface photoelectric absorption and some compounded filament attenuation occurring along the propagation direction. At higher energies, Compton scattering became dominant, while the impact of the pad Z or thickness decreased as shown in Figure 14 and Figure 15.

#### 3.4.2. Electron Irradiation

**Figure 16:**
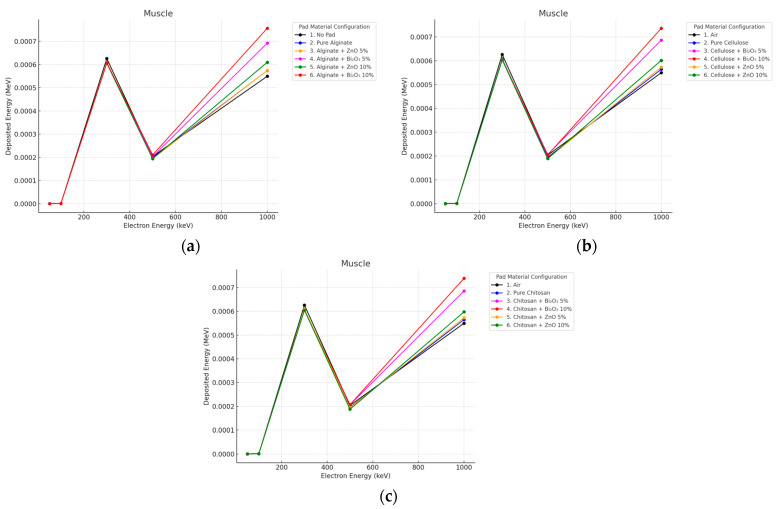
Electron energy deposition profiles in a (muscle) under different polymer pad configurations at 2 mm thickness. (**a**) (Alginate-based). (**b**) (Cellulose-based). (**c**) (Chitosan-based).

**Figure 17:**
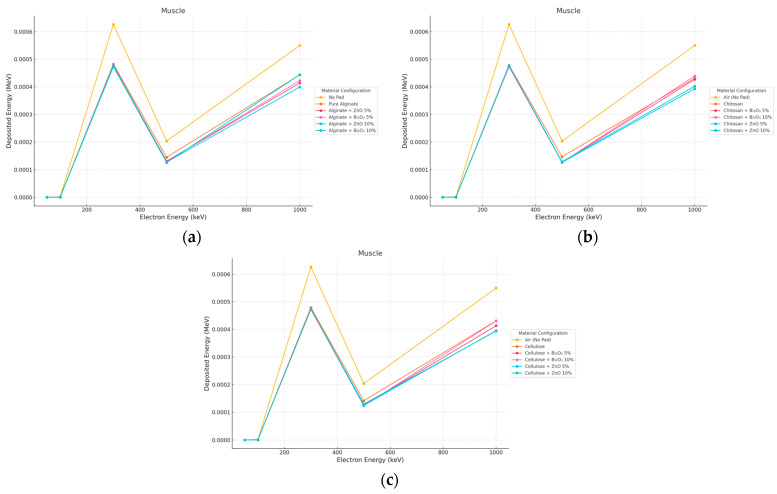
Electron energy deposition profiles in a (muscle) under different polymer pad configurations at 10 mm thickness. (**a**) (Alginate-based). (**b**) (Cellulose-based). (**c**) (Chitosan-based).

The electron dose in the muscle showed a Bragg-like curve, with maximum deposition occurring at ∼300 keV and sharpening reduction at higher energies as a result of overshoot beyond the muscle depth. Notably, for Bi_2_O_3_ 10% at 2 mm, unexpected deep dose increases, probably due to forward secondary electron scattering, were observed and in 10 mm muscle, the dose was consistently reduced by ZnO 10% more than Bi_2_O_3_, serving to confirm the stability of ZnO at high energies as shown in Figure 16 and Figure 17.

The deep muscle dose enhancement observed for Bi_2_O_3_ 10% pads at certain high electron energies can be attributed to the generation of forward-directed secondary electrons and bremsstrahlung photons within the high-Z filler. Dense materials like Bi_2_O_3_ increase the likelihood of inelastic collisions and high-energy photon emission, which can penetrate beyond superficial tissues and deposit energy in deeper layers.

For photon beams, the enhanced epidermal dose for Bi_2_O_3_-loaded pads is explained by the strong Z^3^/E^3^ dependence of the photoelectric effect. Secondary photoelectrons produced within the pad contribute to this increase. At higher photon energies, Compton scattering dominates, making interaction probabilities less sensitive to atomic number and resulting in reduced dose enhancement or suppression.

### 3.5. Heatmap Analysis and Thickness Effects

**Figure 18:**
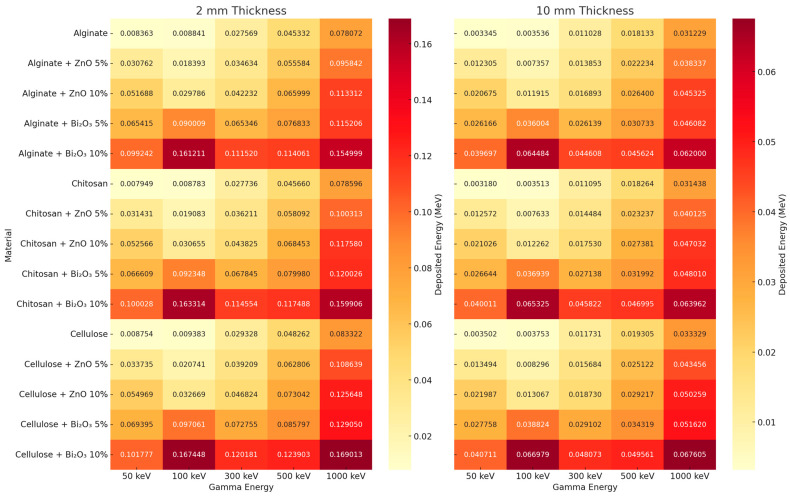
Heat map of energy deposition inside the radiotherapy pad when irradiated by photon beams.

Figure 18 Shows a heatmap comparison that illustrates the deposited gamma energy (MeV) in the pad layer across different polymer materials (Alginate, Chitosan, Cellulose), filler types (ZnO, Bi_2_O_3_), and concentrations (5% and 10%), at two pad thicknesses: 2 mm (left) and 10 mm (right), over a range of gamma energies from 50 keV to 1000 keV.

At 2 mm thickness, energy deposition increases with gamma energy, with the highest values consistently recorded in Bi_2_O_3_-doped polymers, especially at 10% loading. Among these, Cellulose + Bi_2_O_3_ 10% exhibits the maximum deposited energy across nearly all energies, peaking at 0.169 MeV at 1000 keV. This confirms that higher-Z fillers (Bi_2_O_3_) significantly enhance photon attenuation due to the photoelectric effect dominance at lower energies and Compton interactions at higher energies.

At 10 mm thickness, overall deposited energy values are lower, reflecting stronger shielding and more photon attenuation before energy reaches the pad’s inner region. Again, Bi_2_O_3_ composites maintain superior performance, but the energy values are less concentrated due to increased depth. Cellulose + Bi_2_O_3_ 10% still leads with 0.0676 MeV at 1000 keV, reinforcing that both filler concentration and pad thickness play synergistic roles in enhancing radiation absorption.

In summary, the plot demonstrates that Bi_2_O_3_ is more effective than ZnO, and Cellulose outperforms Alginate and Chitosan in enhancing energy deposition, especially when used with higher filler ratios and thicker pads, making it the most promising candidate for radiotherapy pad applications.

**Figure 19:**
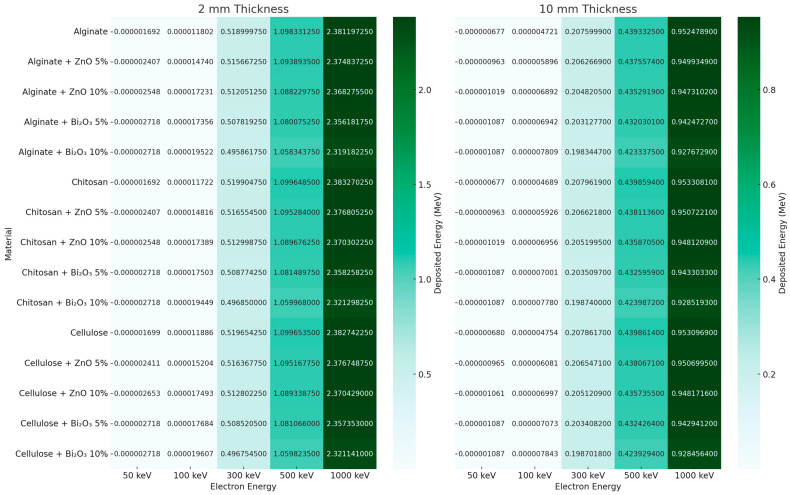
Heat map of energy deposition inside the radiotherapy pad when irradiated by electron beams.

Figure 19 presents the electron energy deposition (MeV) in the pad layer of various polymer materials (Alginate, Chitosan, Cellulose) with and without high-Z fillers (ZnO, Bi_2_O_3_ at 5% and 10%) across electron energies from 50 keV to 1000 keV, at 2 mm (left) and 10 mm (right) thicknesses.

At 2 mm thickness, the energy deposition increases significantly with electron energy, with peak values observed at 1000 keV for all materials. The presence of Bi_2_O_3_ slightly decreases the deposited energy compared to pure polymers at higher energies, due to enhanced backscattering and reduced electron penetration depth. For instance, pure Cellulose shows a maximum deposition of 2.38 MeV, while Cellulose + Bi_2_O_3_ 10% yields 2.32 MeV. This indicates that high-Z fillers increase stopping power, reducing transmission of electrons through the pad.

At 10 mm thickness, the overall deposited energy decreases across all energies and materials, reflecting more electron attenuation and absorption within the thicker pads. Here, the range of deposited energy is narrower (e.g., 0.92–0.95 MeV at 1000 keV), and differences between materials become less pronounced. Cellulose and Chitosan pads, regardless of filler, show slightly better energy deposition retention, with Cellulose + ZnO 5% maintaining the highest value (0.9507 MeV) at 1000 keV.

Overall, the results show that polymer type, filler concentration, and thickness all affect electron energy deposition, with thicker pads and Bi_2_O_3_ fillers providing more electron attenuation, though with lower deposited energy in the pad layer due to reduced penetration and increased scattering.

## 4. Discussion

Electron irradiation showed a typical Bragg-like dose distribution, with energy peaking in intermediate tissues (dermis or subcut) and falling off at greater depths or at higher energies. Without shielding, this resulted in significant dose deposition through all depths—most at 300–500 keV, where electrons travel the deepest before stopping. Integrating 10 mm hydrogel pads resulted in near-complete dose suppression in the epidermis, dermis, and subcut tissues, and only residual energies deposited in muscle were observed in a few configurations. The key attenuation mechanisms were collisional energy loss and multiple scattering, which depend on material density and apparent thickness. Atomic number (Z) had a minor effect, mainly affecting electron stopping power and bremsstrahlung production.

Bi_2_O_3_ exhibited enhanced energy dissipation and scattering; thus, deep dose suppression was achieved, where ZnO had less risk of backscatter or deep-layer dose amplification and was comparable at a high concentration (10%).

The Bi_2_O_3_ 10% differed, as these pads occasionally gave a high dose at high energies to the muscle. This counterintuitive finding is due to the scattered secondary electrons, a well-known effect for dense high-Z materials that can redirect energy into deeper tissues.

As for photon irradiation, the dose was depth and energy dependent, which was seen from the penetration and low photon interaction probability. The main physical processes, the photoelectric effect at low energies (50–300 keV) and Compton scattering at high energies (≥500 keV), were significantly dependent on atomic number. The Z3/E3 dependence of the photoelectric effect resulted in substantial dose enhancement in the epidermis due to Bi_2_O_3_-doped pads, particularly at 100 keV and below. This was attributable to secondary photoelectrons produced in the pad; these transmit energy to the skin. At higher photon energies, Compton scattering dominated, which led to improved dose uniformity in tissues and decreased importance of the Z factor; the distinction between ZnO and Bi_2_O_3_ decreased, and the pure polymers performed similarly to that of their doped forms.

From another point of view, each base polymer, Alginate, Chitosan, and Cellulose, exhibited the diverse effects of radiation associated with their own physical, chemical, and biomechanical properties. Alginate is soft and hydrophilic and can conform to tissue surfaces with minimum effort; it works extremely well in electron shielding, both in pure and doped forms, especially at a thickness of 10 mm. As in photon shielding, it has less performance as the surface dose sharply increases. This means it is best used for surface shielding in electron therapy [7,8,9,10,11,12]. Chitosan is semi-rigid, has mechanical stability, and has biocompatibility. It has excellent electron shielding, and the Bi_2_O_3_ 10% resulted in nearly zero dose in all layers. While for photon irradiation, the energy deposition behavior was relatively the same with ZnO 10%, the Bi_2_O_3_ gave a smooth response and low surface dose amplification. These results show that it can be used in those therapies requiring greater depth of electron attenuation and structural integration [17,18,19,20,21,22]. Cellulose has the highest density among the studied polymers and dominated all others in muscle and subcut dose reduction, especially with Bi_2_O_3_ 10%, but it is a little less effective for surface protection than Chitosan, excellent overall, and best for high-energy gamma shielding or mixed therapy modalities [19,20,21,22].

Our results highlight a paradox for Bi_2_O_3_-loaded pads in the 50–300 keV photon range: enhanced surface dose due to photoelectric secondaries. Clinically, this could be mitigated by reducing the Bi_2_O_3_ concentration, adding a thin low-Z overlayer above the Bi_2_O_3_ composite, or selecting ZnO in scenarios where epidermal sparing is paramount. ZnO’s lower Z reduces both photon–electron conversion and electron backscatter, at the cost of somewhat reduced deep photon attenuation. From a materials perspective, ZnO composites are also lighter, more flexible, and highly biocompatible, making them attractive for patient comfort and wound-contact applications. Conversely, Bi_2_O_3_ remains advantageous where deep-layer attenuation is the primary goal, particularly for photons in the photoelectric and mixed interaction regimes.

While Bi_2_O_3_ composites demonstrated superior photon attenuation, their clinical use should be optimized to avoid surface dose enhancement in the low-energy photon range and potential bremsstrahlung generation during electron therapy. Practical strategies include lowering filler concentration, adding a thin low-Z overlayer, or substituting with ZnO or other high-Z oxides with more favorable scattering profiles. ZnO offers lower deep-layer dose perturbation and higher mechanical flexibility, making it suitable for mixed photon–electron scenarios. Conversely, Bi_2_O_3_ remains advantageous where deep-layer attenuation is the primary goal, particularly for photons in the photoelectric and mixed interaction scenarios.

Further work should quantify and experimentally verify bremsstrahlung contributions from high-Z fillers under therapeutic electron beams to guide safe clinical implementation.

Beyond the two fillers examined here, other high-Z oxides such as tungsten oxide (WO_3_) and tantalum oxide (Ta_2_O_5_) have demonstrated high photon attenuation efficiency and good chemical stability [32,33,34]. These materials have been incorporated into polymer matrices for radiological shielding in both medical and industrial contexts. Future studies could explore these alternatives in hydrogel systems to expand the range of tunable shielding–mechanical–biocompatibility trade-offs available for clinical customization.

Table 3 summarizes actionable pad selections by clinical goal, following the trends reported in the results.

## 5. Conclusions

In this study we provided a full Monte Carlo simulation-based investigation of the radiation shielding efficiency of biocompatible hydrogel pads developed with Alginate, Chitosan, and Cellulose, formulated with and without high-Z fillers (ZnO and Bi_2_O_3_) at 5% and 10%. Energy deposition was calculated in a multilayer tissue equivalent phantom (epidermis, dermis, subcutaneous, and muscle) using the Geant4 toolkit for different gamma and electron beams based on a typical clinical energy range (50–1000 keV). The results present new information on depth-dependent responses of radiation-matter interactions for soft polymeric shielding and practical design guidelines for clinical applications. The study focuses on the use of hydrogel-based shields in external beam radiotherapy and surface protection applications with customized material and filler configurations with respect to beam type, energy, and tissue depth.

While the present study employed monoenergetic photon and electron beams to clearly isolate the underlying energy-dependent interaction mechanisms, we recognize that clinical beams possess complex energy spectra. Future investigations will incorporate full clinical beam models to directly relate these findings to specific treatment modalities. Likewise, the current use of a flat, layered phantom was intended to match widely used reference geometries in radiotherapy physics research, enabling direct comparison with prior studies. Building on this, future work will expand to anatomically realistic 3D phantoms to evaluate the influence of curvature, anatomical variation, and tissue heterogeneity on shielding performance.

## Figures and Tables

**Table 1:** Composition and characteristics of simulated base hydrogels.

Hydrogel	Density (g/cm^3^)	Polymer Formula	Key Properties
Chitosan	1.05	C_6_H_11_NO_4_	Rigid, antimicrobial, biocompatible
Alginate	1.05	C_6_H_7_NaO_6_	Soft, hydrophilic, moisture-retentive
Cellulose	1.10	C_6_H_10_O_5_	Structurally robust, widely used in biomedicine

**Table 2:** Characteristics of high-Z dopants.

Filler	Density (g/cm^3^)	Chemical Formula	Functionality
Bi_2_O_3_	8.90	Bi_2_O_3_	High photon attenuation efficiency
ZnO	5.60	ZnO	Moderate shielding with improved flexibility

**Table 3:** Practical recommendations for hydrogel radiotherapy pads by clinical goal (derived from simulation results).

Clinical Goal	Recommended Polymer and Filler	Filler (%)
Minimize surface (epidermal) dose in electron therapy	Alginate + ZnO	0–10%
Reduce mid-depth (dermis/subcut) dose in electron therapy	Chitosan or Cellulose + ZnO	5–10%
Improve deep-tissue attenuation (muscle) in photon therapy	Cellulose + Bi_2_O_3_	10%
Balance surface dose control with photon shielding at low energies	Cellulose + ZnO	5–10%
Maintain comfort/weight with moderate shielding	Alginate or Chitosan + ZnO	5%

## Data Availability

The original contributions presented in this study are included in the article. Further inquiries can be directed to the corresponding author.
